# Compassionate use programmes for rare diseases: proposals for actions

**DOI:** 10.1186/1750-1172-7-S2-A28

**Published:** 2012-11-22

**Authors:** François Houÿez, Chantal Bélorgey, Arielle North, Etelka Czondi, Michele Lipucci di Paola

**Affiliations:** 1 The European Organisation for Rare Diseases; 2 Agence Nationale de Sécurité des Médicaments, France; 3 Ancre Consulting, formerly European Medicines Agency; 4 Programmes Coordinator, Sense International, Romania; 5 Associazione Veneta per la Lotta Alla Talassemia

## Aims

Patients’ advocates, regulators and doctors have the constant objective to make the best treatments quickly available to patients. Compassionate use programmes (CUP) as defined by the EU Regulation (EC) № 726/2004 article 83.2 are designed to provide early access before the marketing authorisation (MA) of a medicine. It is necessary to evaluate the achievements of the present legislations on CUP in Europe in order to propose actions that could integrate CUP into an improved orphan drug development model.

## Rationale

The European legislation is not applied on the same way in the Member States (MS) even if in almost all MS a scheme for CUP exists. The experience of the European Medicines Agency, MS and companies is that this lack of harmonisation makes difficult the early access to important new medicines particularly for rare diseases.

## Methods

Series of workshops with all stakeholders and a survey conducted by EURORDIS in 2010-2011 via a questionnaire to 64 holders of a marketing authorisation for an orphan medicinal product helped analysing current practices across Europe, experience at the European Medicines Agency’s (EMA) level, and how CUPs are inserted in the general development of an orphan medicinal product.

## Results

The survey showed the obstacles in the implementation of such programmes, and also their benefits. By enlarging the number of patients exposed to a new product, CUPs can expand the number of exposed patients in safety databases by 320%. Nine products with a CUP were reviewed, some programmes starting early in the development of the product, others lately. Only France could provide all 9 products on a compassionate basis, while 3 countries could provide a programme for 5-6 products, 5 countries for 3-4 products, 23 countries to 1-2 products, and 10 did not provide any product on a compassionate basis.

The French Temporary Use Authorisation system (A.T.U) illustrates the benefit for public health CUPs can represent: combining early access to drugs for patients, close control by the competent authority of the use of new drugs and appropriate information of stakeholders. France could provide 72% of the 64 authorised orphan medicinal products on a compassionate basis in average 35 months prior to the MA.

## Main conclusions

This work led to an initiative to improve information and transparency about CUPs in Europe, and also to propose good practices in this domain. In order to improve the situation it is proposed to pursue the dialogue with MS and companies and to set up a “Facilitation group” between MS in order to exchange information and to build up on common experiences.

